# Editorial: Recent advances in hazelnut (*Corylus* spp.)

**DOI:** 10.3389/fpls.2022.1120595

**Published:** 2023-01-04

**Authors:** Valerio Cristofori, Roberto Botta, Mercè Rovira, Thomas J. Molnar, Shawn A. Mehlenbacher

**Affiliations:** ^1^ Department of Agricultural and Forest Sciences (DAFNE), University of Tuscia, Viterbo, Italy; ^2^ Department of Agricultural, Forest and Food Sciences (DISAFA), University of Turin, Grugliasco, Italy; ^3^ Institute of Agrifood Research and Technology ( Institut de Recerca i Tecnologia Agroalimentàries (IRTA) - Mas Bové), Constantí, Spain; ^4^ Department of Plant Biology, School of Environmental and Biological Sciences, Rutgers University, New Brunswick, NJ, United States; ^5^ Department of Horticulture, Oregon State University, Corvallis, OR, United States

**Keywords:** *Corylus* spp., breeding programs, cultivar choice, precision agriculture, climatic adaptation

The European hazelnut (*Corylus avellana* L.) of the genus *Corylus* is a major species of interest for nutritional use, and its nuts are widely used worldwide in the chocolate, confectionery, and bakery industries. Other *Corylus* species, including *C. americana*, *C. heterophylla*, and *C. colurna*, are of local interest for commercial uses and provide important genetic resources for breeding. The global production of hazelnut has increased since the beginning of the last century, especially in the last 10 years in response to the demands of the confectionery industry, which processes about 90% of the harvested nuts. Cultivation of this nut has expanded in several traditional countries as well as in new areas, including Chile, South Africa, and Australia.

Therefore, this Research Topic devoted to European hazelnut and its related species has been designed with the aim of assembling an effective-knowledge platform based on recent research carried out in the *Corylus* spp. sector.

The Research Topic brings together some of the latest research outputs in hazelnut cultivation, genetic resources, and post-harvest processing, thanks to the excellent response to the call which resulted in the publication of 25 original articles. We found the edition and selection of articles for this book very inspiring and rewarding. We also thank the editorial staff and reviewers for their efforts and help during the process.

The scientific contributions are briefly discussed below, grouped into seven sections according to the main topics proposed during the Research Topic launch.

## Advances in hazelnut breeding methods, new releases from breeding programs, advances in genome sequencing, and development of markers for traits of interest

In this section mainly devoted to breeding and genome sequencing, four original articles were grouped and published.

In the first article, titled “*Unraveling Genetic Diversity Amongst European Hazelnut (Corylus avellana L.) Varieties in Turkey*” (Oztolan-Erol et al.), the authors discuss the genetic variation among hazelnut varieties defining variety-specific and disease resistance-associated alleles, prone to facilitating hazelnut breeding in Turkey. This study provides suitable molecular markers to establish a protection program for the most commercially valuable hazelnut varieties, such as “Tombul”, which does not yet present an elite line.

The second contribution focuses on “*New Sources of Eastern Filbert Blight Resistance and Simple Sequence Repeat Markers on Linkage Group 6 in Hazelnut (Corylus avellana L.)*” (Koma et al.). The authors discuss the genetic resistance to control the disease Eastern Filbert Blight caused by *Anisogramma anomala* (Peck) E. Müller. In this study, new simple sequence repeat (SSR) markers were developed for the resistance region on LG6, and new sources of resistance were investigated. In total, 42 new SSR markers were developed from four contigs in the genome sequence of cv ‘Jefferson’ released by the Oregon State University breeding program. These new LG6 resistance sources and SSR markers may be useful in breeding new cultivars.

The third article presents the “*Genome-Wide Identification of the ARF Gene Family and ARF3 Target Genes Regulating Ovary Initiation in Hazel via ChIP Sequencing*” (Wei et al.). The research discusses the regulation of auxin in ovary development, which is thought to be related to auxin response factors (ARFs). The authors suggest that ChARF3 (*C. heterophylla* ARF3) may regulate ovary initiation and ovule development by mediating genes related to auxin biosynthesis and transport, cell division and proliferation, and flower and fruit development. This study provides new insights into the molecular mechanism of hazelnut nut formation.

The fourth contribution is titled “*Mapping the Genetic Regions Responsible for Key Phenology-Related Traits in the European Hazelnut*” (Valentini et al.). The authors discuss a rational approach for mapping QTL for phenology-related traits, such as the time of male and female flowering, dichogamy, and the period required for nut maturation, with the aim of identifying quantitative trait loci (QTL) and specific genes associated with plant phenology. Overall, 71 QTLs were detected, of which 20 were identified as contributing to the time of male flowering, 15 to the time of female flowering, 25 to dichogamy, and 11 to the time of nut maturity.

## Genetic resources for potential expansion of hazelnut production to new locations

In the second section, a further four original articles were grouped as follows.

The first contribution is titled “*Chromosome-Level Genome Assembly and Hazel Omics Database Construction Provides Insights into Unsaturated Fatty Acid Synthesis and Cold Resistance in Hazelnut (Corylus heterophylla)*” (Liu et al.). The authors discuss the unclear mechanism underlying the adaptation of *C. heterophylla* to extremely low temperatures. Through genome evolution analysis, 17 expanded genes were identified, which were found to be significantly enriched in the unsaturated fatty acid biosynthesis pathway (ko01040). It was deduced that the expansion of these genes may promote a high unsaturated fatty acid content in kernels and improve the adaptability of *C. heterophylla* to the cold climate of north-eastern China.

The second article, entitled “*Is It Possible to Produce Certified Hazelnut Plant Material in Sicily? Identification and Recovery of Nebrodi Genetic Resources, in vitro Establishment, and Innovative Sanitation Technique from Apple Mosaic Virus*” (Yahyaoui et al.), discusses the sanitation of eight Sicilian cultivars to obtain plants free from the Apple Mosaic Virus affecting European hazelnut. The authors investigated the possibility of establishing *in vitro* true-to-type and virus-free hazelnut plantlets *via* the encapsulation technology of apexes.

A third article, titled “*Stigmatic Transcriptome Analysis of Self-Incompatible and Compatible Pollination in Corylus heterophylla Fisch. x Corylus avellana L.*” (Hou et al.), highlights the molecular mechanism of saprophytic self-incompatibility (SSI) in some *Corylus* spp. that remains currently largely unknown. From self-pollination experiments (‘Dawei’ _ ‘Dawei’) and cross-pollination experiments (‘Dawei’ _ ‘Liaozhen No. 7’) and later an RNA-Seq analysis, the mechanism of pollen-stigma interactions was investigated to identify genes that may be responsible for SSI, discovering 19,163 up- and 13,314 downregulated genes, some of these potentially involved in pollen stigma interactions and SSI mechanisms.

The fourth contribution is titled “*Variation of Morphological, Agronomic and Chemical Composition Traits of Local Hazelnuts Collected in Northern Spain*” (Negrillo et al.). The authors discuss phenotypic variation for phenological and morphological traits, chemical composition, and investigated a field collection of 41 local and 17 non-local accessions in Villaviciosa (Spain). A large degree of variation for most morphological and phenological traits, except nut maturity date, was revealed.

## Recent findings in the propagation of *Corylus* spp., including micropropagation

The third section grouped two original articles.

In the first contribution, titled “*Advances in Nursery Production of Hazelnut Plants in Serbia-Successful Grafting of Different Corylus avellana L. Cultivars and Clones onto Corylus colurna L. Rootstock*” (Bijelic et al.), the authors discuss the grafting affinity of the main Italian cultivars (“Tonda Gentile Romana”, “Tonda di Giffoni”, and “Tonda Gentile delle Langhe”) and their clones onto Turkish filbert seedlings (*C. colurna* L.) to be used as non-suckering rootstocks, evaluating possible differences in the quality of the obtained planting material. The results revealed that the chosen hazelnut cultivars and clones exhibited excellent grafting success rates, with the highest grafting success by clone “AD17”.

The second article is titled “*Agronomical and Physiological Behavior of Spanish Hazelnut Selection “Negret-N9” Grafted on Non-suckering Rootstocks*” (Rovira et al.). The authors discuss the grafting effect of the cultivar “Negret N-9” onto four low sucker emission clonal rootstocks (Dundee, Newberg, Tonda Bianca, IRTA MB-69) in comparison with the self-rooted plants of “Negret N-9” selected as control. The results showed that clonal rootstocks had a strong influence on the vigor and yield of “Negret N-9.” Physiological traits indicated a higher overall performance with “Dundee” rootstocks. Further, “Dundee,” “Newberg,” and “IRTA MB-69” rootstocks showed very low emission of suckers.

## Advances in knowledge of hazelnut pests, diseases, and control methods

The section grouped four more original articles, mainly concerning phytopathological microorganisms such as bacteria and fungi.

The first contribution, titled “*Draft Genome Sequence of a New Fusarium Isolate Belonging to Fusarium tricinctum Species Complex Collected from Hazelnut in Central Italy*” (Turco et al.), discusses the typical symptomatology (brown-grayish spots at the bottom of the nuts progressing upward to the apex) associated with the Nut Gray Necrosis syndrome, likely caused by *Fusarium lateritium*. To increase knowledge of this fungal pathogen, whole-genome sequencing of a strain isolated from symptomatic hazelnuts was performed. The following phylogenetic and comparative genomics analysis suggested that the isolate collected in central Italy is induced by the *F. tricinctum* species complex rather than *F. lateritium* one.

The second article is titled “Susceptibility of some *Corylus avellana* L. Cultivars to *Xanthomonas arboricola* pv. *corylina*” (Webber et al.). The authors discuss the susceptibility of hazelnut cultivars to *Xanthomonas arboricola* pv. *corylina* (Xac). Two inoculation protocols of Xac were investigated in two different hazelnut cultivation environments to assess cultivar susceptibility: *in vitro* tissue culture under sterile and controlled conditions tested on five cultivars, and *in vivo* potted tree conditions tested on seven cultivars. Under *in vitro* conditions, severe bacterial blight symptoms were noticed, consistent with those seen in the field, without significant differences in the susceptibility from the cultivar tested. Under *in vivo* conditions, the proportion of necrotic buds was significantly higher in “Jefferson” and “Dorris” compared with the other tested cultivars, including “Barcelona.”

The third contribution, titled “*Molecular Characterization of Diaporthe Species Associated with Hazelnut Defects*” (Arciuolo et al.), is focused on which fungal species are present in defective hazelnuts from Turkey, including the role of *Diaporthe* spp. Seven Turkish hazelnut orchards differently located were tested and several genera were isolated, with *Diaporthe* spp. being among the most prevalent, highlighting that *Diaporthe* strains can be grouped into seven distinct clades, with the majority of Turkish strains (95%) being placed into a single clade related to *D. eres*.

The last article of this section is titled “*Characteristics of the Fungal Communities and Co-occurrence Networks in Hazelnut Tree Root Endospheres and Rhizosphere Soil*” (Ma et al.). The authors determined the fungal communities in the root endosphere and rhizosphere soil of four hazelnut species by DNA sequencing. Two-factor correlation network analysis and linear regression analysis showed that the total organic carbon was the main environmental factor affecting the fungal communities.

## Understanding the eco-physiological behavior of European hazelnut and its relatives

In this section, five original articles were grouped and published.

The first contribution is titled “*Effect of Photo-Selective Shade Nets on Pollination Process and Nut Development of Corylus avellana L.*” (Guastella et al.). The authors discuss the effects of photoselective nets on the pollination process and nut development of hazelnut in South Africa. Mature hazelnut trees were maintained under netting and compared with the ones in open fields. Results showed differences in pollen tube growth, the timing between treatments, and even differences in ovule abortion. The shade nets influenced the pollen tube growth and the nut development, principally due to micro-climate modification, and a higher rate of abortion was detected in open fields compared to the plants under netting.

In the second article, titled “*Hazelnut Pollen Phenotyping Using Label-Free Impedance Flow Cytometry*” (Ascari et al.), the authors discuss the use of Impedance Flow Cytometry (IFC) to characterize hazelnut pollen viability during its dehiscence. IFC was validated *via* dye exclusion in microscopy and employed to follow pollen hydration over time to define the best pre-hydration treatment for pollen viability evaluation and test hazelnut pollen viability and sterility on 33 cultivars and two wildtypes grown in a collection field located in central Italy. Pollen sterility rate varied greatly among hazelnut accessions, with one main group of highly sterile cultivars and a second group, comprising also the wild genotypes, producing good quality pollen.

The third contribution is titled “*Assessment of Canopy Conductance Responses to Vapor Pressure Deficit in Eight Hazelnut Orchards Across Continents*” (Pasqualotto et al.). The article treated the asses of the tree conductance responses to Vapor Pressure Deficit (VPD), as a key step for modeling plant performances and productivity under future environmental conditions, especially when trees are cultivated outside their suited areas for soil and climate conditions. The results can be used for defining suitability maps based on average VPD conditions, facilitating the correct identification of the potentially most productive sites.

The fourth article, titled “*Combined Spraying of Boron and Zinc During Fruit Set and Premature Stage Improves Yield and Fruit Quality of European Hazelnut* cv. *Tonda di Giffoni*” (Meriño-Gergichevich et al.), refers to the efficiency of combined boron (B) and zinc (Zn) spraying in relation to European hazelnut phenological stages. The study carried out on 9-year-old trees of “Tonda di Giffoni” highlighted moderate and partialized rates of B and Zn and the time of their application contribute to improving yield and nut traits in the crop.

The fifth article is titled “*Effects of Living Cover on the Soil Microbial Communities and Ecosystem Functions of Hazelnut Orchards*” (Ma et al.). The authors discuss the differences observed between soils with living cover treatments with *Vulpia myuros* and soils without cover treatments, analyzing changes in soil properties, microorganisms, and microbial functions. According to their findings, the application of a living cover with *V. myuros* showed a favorable regulatory influence on soil properties, microbial communities, and microbial function.

## New prototypes and precision farming applications for sustainable intensification of production in hazelnut orchards

In this section, two original articles were submitted and published.

An article titled “*The HADES Yield Prediction System - A Case Study on the Turkish Hazelnut Sector*” (Bregaglio et al.) discusses the application of machine learning for yield prediction in Turkish environments, through a system called HADES (HAzelnut yielD forEcaSt). HADES paves the way for a next-generation yield prediction system, to deliver timely and robust information and enhance the sustainability of the hazelnut sector across the globe.

The second contribution, titled “Rotten Hazelnuts Prediction *via* Simulation Modeling - A Case Study on the Turkish Hazelnut Sector” (Valeriano et al.), is focused on the quality defects of the nuts, which comprise changes in morphology and taste, highlighting that their intensity mainly depends on seasonal environmental conditions. In Turkey, a rotten defect forecasting model was proposed, and the results confirmed that the rotten defect is strictly dependent on precipitation amount and timing, as well as plant susceptibility being crucial to triggering fungal infections.

## Advances in industrial processing, nutritional value, and the health benefits of hazelnuts

Four original articles were grouped in the last section according to their main treated topics.

The first contribution is titled “*Corylus avellana: A Source of Diarylheptanoids with a-Glucosidase Inhibitory Activity Evaluated by in vitro and in silico Studies*” (Masullo et al.). The authors discuss the biological properties of certain cyclic diarylheptanoids, namely giffonins, a class of natural products isolated from Italian cv. Tonda di Giffoni. The inhibitory effects of diaryletanoids isolated from *C. avellana* by-products against a-glucosidase enzyme were evaluated, promoting the hazelnut leaves as a prospective source of bioactive diarylheptanoids, as a prospective source of bioactive diarylheptanoids for the development of functional ingredients for treating diabetes.

The second article titled “Corylus avellana L. Aroma Blueprint: Potent Odorants Signatures in the Volatilome of High-Quality Hazelnuts” (Squara et al), is focused on volatilome of hazelnuts that encrypts information about phenotype expression as a function of cultivar/origin, post-harvest practices, and their impact on primary metabolome, storage conditions, shelf-life, spoilage, and quality deterioration. Within the bulk of detectable volatiles, few of them play a key role in defining distinctive aroma (i.e., aroma blueprint) and conferring characteristic hedonic profiles.

The third article of the section, titled “*Corylus avellana L. Natural Signature: Chiral Recognition of Selected Informative Components in the Volatilome of High-Quality Hazelnuts*” (Stilo et al.), treated the enantiomeric composition of a large set of chiral compounds within the complex volatilome of European hazelnut belonging to different cultivars, harvested in different geographical areas. The results showed that chiral compounds have diagnostic distribution patterns within hazelnut volatilome with cultivar and harvest region playing the major roles.

The last contribution of the section, titled “*Kernel Nutrient Composition and Antioxidant Ability of Corylus* spp. *in China*” (Jiang et al.), discusses the nutritional quality of hazelnuts from Chinese *Corylus* species. Four wild *Corylus* spp. (*C. heterophylla* Fisch., *C. mandshurica* Maxim., *C. kweichowensis* Hu., and *C. yunnanensis* Franch.) originating from China and the Chinese hybrid hazelnut cultivar ‘Dawei’ (*Corylus heterophylla* Fisch. x *C. avellana* L.) were used to analyze the basic nutritional composition and antioxidant ability. Compared to the four wild hazelnut kernels, ‘Dawei’ had higher oil, oleic acid, a-tocopherol, and sugar content. Overall, as discussed in the article, large differences emerged in the nutritional composition of the different *Corylus* species analyzed.

In summary, the original articles published in this Research Topic represent some of the latest and most promising research outputs linked to science and technology applied to the *Corylus* spp. We thank all authors of the Research Topic, hoping that the research findings here collected will be useful and guide the future technical and scientific activities of breeders, geneticists, eco-physiologists, horticulturists, plant pathologists, and agricultural engineers involved in the hazelnut sector.

## Author contributions

All authors listed have made a substantial, direct, and intellectual contribution to the work and approved it for publication.

